# Bladder Cancer Sample Handling and Reporting: Pathologist's Point of View

**DOI:** 10.3389/fsurg.2021.754741

**Published:** 2021-12-02

**Authors:** Roberta Mazzucchelli, Daniela Marzioni, Giovanni Tossetta, Laura Pepi, Rodolfo Montironi

**Affiliations:** ^1^Section of Pathological Anatomy, Department of Biomedical Sciences and Public Health, Università Politecnica delle Marche, Ancona, Italy; ^2^Departement of Experimental and Clinical Medicine, Università Politecnica delle Marche, Ancona, Italy

**Keywords:** bladder, carcinoma, cystectomy, staging, handling, reporting

## Abstract

The aim of this narrative review is to provide adequate information on handling and reporting of the bladder cancer samples to improve the closely collaboration between pathologists and urologists. The main (but not exclusive) research tool used was PubMed and 87 references were selected and quoted in the text. We have considered handling of biopsies, transurethral resection (TUR), and cystectomy specimens to summarize the different methods of sampling and the related issues. Moreover, we considered and discussed the main prognostic factors, such as histological tumor type, grade, and stage of bladder cancer, that should be described in the pathological report. In addition, critical issues encountered in the interpretation of histological samples were discussed.

## Introduction

A close collaboration between urologist and pathologist is essential for accurate diagnosis and management of patient with bladder cancer. The decision-making for the treatment of bladder cancer depends on both quality of surgical specimen and accuracy of the pathological diagnosis. The precise description of clinical history and an adequate urological information, such as bladder lesion cystoscopic or tomographic scan appearance, timing, surgical or therapeutic procedure performed allow pathologist to decide the best approach in handling and processing the surgical specimens, so as to obtain an accurate pathology report (1–5). The reports can present some differences on the basis of the surgical specimens: for example, report on transurethral resection (TUR) specimens supplies the main information that determines subsequent patient management, such as re-TUR or radical treatment, while report on cystectomy may have an effect on further adjuvant chemo/radiotherapy or appropriate surveillance. In this review, we have considered handling of biopsies, TUR, and cystectomy specimens to summarize the different methods of sampling and the related issues. Moreover, the main prognostic factors, such as histological tumor type, grade, and stage of bladder cancer, that should be described in the pathological report were considered and discussed to make understandable terminology and histopathological problems to urologists (6, 7). The aim of this narrative review was to provide practical points for pathologists and urologists concerning the above described trans-disciplinary topics. The main (but not exclusive) research tool was PubMed. The key words used were “bladder cancer or bladder carcinoma,” in addition to various combinations of stages, grades, variants, lympho-vascular invasion, handling, pathological report, and histopathological report. Collateral research included “histochemistry and smoothelin” and “histoanatomic variance and bladder.” The cited articles were mostly published between 2009 and 2021. A number of 87 references were selected and quoted in the text. The data were organized in chapters reflecting the current status of bladder cancer handling and reporting.

## Clinical Information

The urologists play a main role in uropathology practice not only as responsible for providing adequate tissue samples for pathological evaluation but also giving useful clinical information to the pathologist to decide the best approach in handling and processing the surgical specimens and draw up an accurate pathology report (1, 3–5).

The urologist should indicate:

- demographic information and clinical history of the patient, bladder cytology if present, whether it is the first presentation of the tumor and if not, details of previous resection;
- the cystoscopic appearance of bladder mucosa and indicate number, size, location of the tumor/s, the morphological features of the lesion: papillary, solid, or ulcerate;
- the state of remaining mucosa if further biopsies were performed;
- if previous radiotherapy to the bladder or to adjacent organ were performed;
- if after the first transurethral resection of bladder tumor (TURBT) local treatments, such as bacillus Calmette-Guérin (BCG) or Mitomycin C intravesical instillation, were performed.

This information is necessary for a correct evaluation of urothelium because the treatments can have an impact on tumor morphology and on normal-looking urothelium in the samples obtained from both re-TURBT or cystectomy (7).

In case of cystectomy, the urologist should provide further information, such as (2, 3):

- information about previous surgical treatments, location, and pathological diagnosis of bladder lesion/s;
- cystoscopic appearance of the bladder mucosa;
- tomographic scan or MRI of the bladder (if performed) to better compare them with macroscopic appearance of the specimen;
- information concerning neoadjuvant chemotherapy, location, number, and size of the lesion/s presents in bladder before therapy to avoid the difficulties in identifying the tumor/s.

## Specimen Handlings

### Biopsy

Biopsies of the bladder can be taken through cystoscope using cold cup forceps, diathermy forceps, or small diathermy loop (8). Biopsy specimen obtained by cold cup forceps does not show artifacts because a Bugbee electrode is used later to cauterize the urothelial defect (9). Tissue bladder biopsy may be obtained using a resectoscope but this procedure is more invasive and a biopsy specimen can show altered histologic characteristics secondary to the effects of electrical coagulation of tissue (8).

The bladder cold cup biopsy is usually 2–3 mm in diameter, it could contain up to the superficial part of muscularis propria (MP) depending on anatomical part of bladder and on operator skill. The biopsy specimens should be wholly paraffin embedded for histological examination. The biopsy specimens can show small papillary neoplasms, erythematous, or velvet area of urothelium that can represent carcinoma *in situ* (CIS) and/or inflammation. Cold cup biopsy mapping of normal-looking mucosa is not in routine use but this approach is recommended for patients with positive urine cytology and negative cystoscopy or a history of high grade non muscle invasive bladder cancer or in tumors with non-papillary appearance (4, 5, 10, 11). To obtain representative mapping of the bladder mucosa, biopsies should be taken from trigone, bladder dome, and right, left, anterior posterior bladder walls. A specimen of urethra may be useful to assess the extension of disease.

Then, these biopsies should be put in separate jars and subsequently paraffin embedded in different blocks. At least tissue sections at three different levels for each biopsy need for histological evaluation. Deeper levels are recommended if the urothelium surface is not wholly visible and to find suitably orientated urothelium (4, 5).

### TUR Specimens

Transurethral resection of bladder tumor is the gold standard for the treatment of non-muscle invasive bladder cancer larger than 6 mm. Tumors ≤ 1 cm as larger size can be resected “en bloc” during TUR procedure (11, 12). En bloc resection is an emerging surgical technique that provides a circumferential incision of the bladder mucosa at a safety margin of few millimeters from the lesion. This technique allows removing the whole tumor, such as the underlying detrusor muscle. Several energy sources are used for this surgical technique, such as monopolar or bipolar current, Holmium and Thulium laser, and hydrodissection (11, 13). Recent studies have demonstrated that “en bloc” resection of bladder tumor (ERBT) should be considered feasible for bladder tumor size of ≤ 3 cm (14–16). The technical limits for ERBT concern mainly the location of the tumors but not their number. In particular, the localization of the tumor at the upper anterior or posterior bladder wall can be considered a limit due to a potential risk of peritoneal damage and the tumor location in bladder dome can be a challenging from a technical point of view (13).

This surgical technique, compared with traditional TUR, provides an intact tumor specimen containing detrusor muscle that allows pathologist to make accurate histopathological evaluation (17). In this type of specimen evaluation of circumferential and deep resection margins must be performed (16).

“En bloc” resection of bladder tumor surgical practice is not yet widely used while TUR remains the surgical procedure more used for non-muscle invasive bladder tumors and for large tumors that can be removed in fragments. The TUR specimens should be weighted in aggregate and processed completely, especially for TUR specimens up to 10 g. When papillary neoplasms are recognizable in these specimens, the number of tissue chips, that shows the lesion and gross tumor size should be recorded and, at least 1 cassette block per cm of tumor, up to 10 blocks, should be sampled initially. For larger specimens, not entirely processed, additional blocks are recommended until complete embedding to rule out histological invasion of either the lamina propria or muscularis propria (4, 5). The European Association of Urology (EAU) guidelines recommend submitting exophytic part of tumor, the tumor base specimens, and the edges of the resection area in separate jars to simplify both the detection of muscularis propria, as a marker of complete local resection, and to evaluate the level of invasion (18).

### Cystectomy Specimens

**Figure 1:**
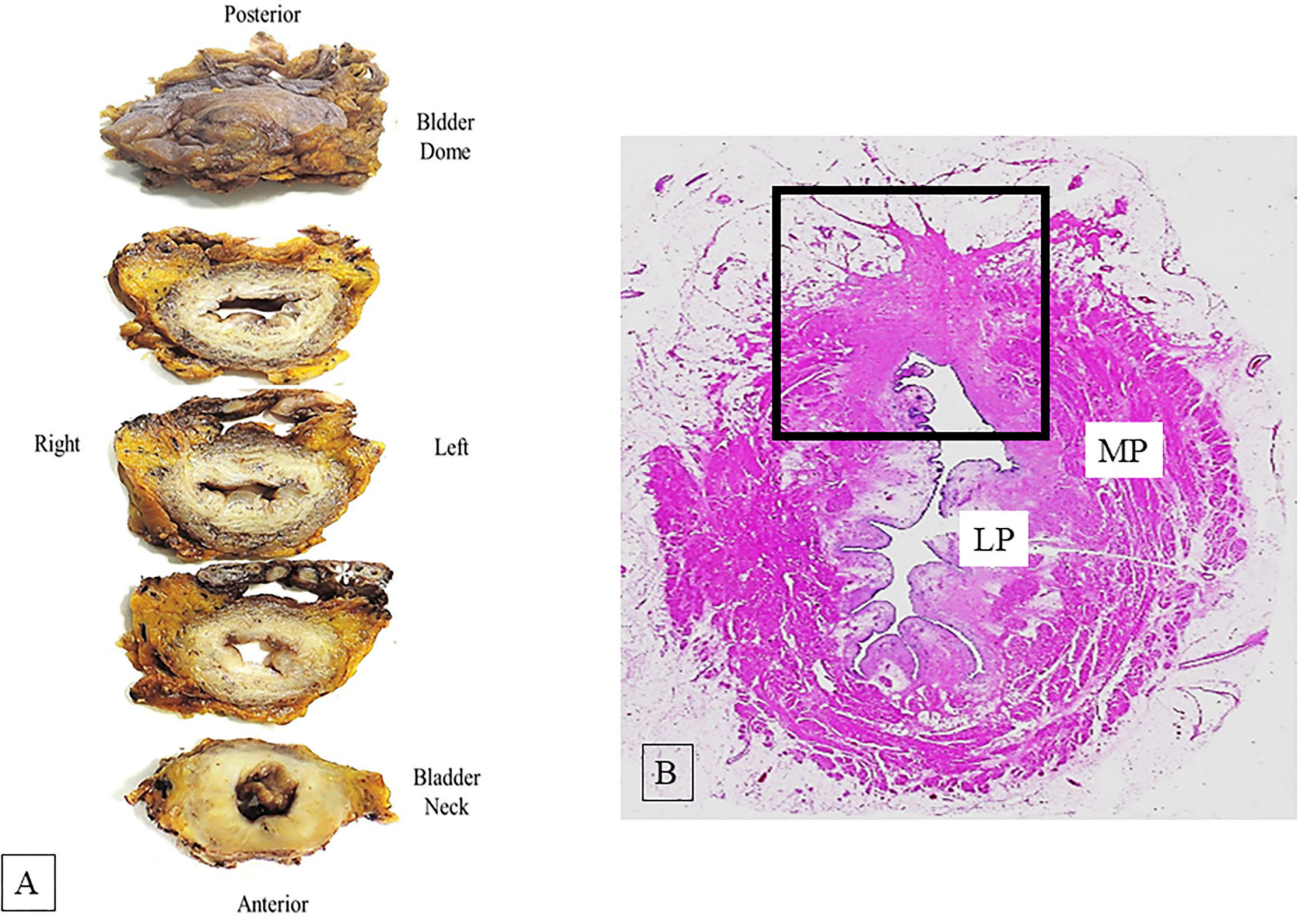
**(A)** Serial dissected bladder transversely from the bladder neck to the dome at 5 mm intervals. The asterisk indicates the seminal vesicles. **(B)** Hematoxylin-eosin whole mount section shows bladder wall architecture; muscularis propria (MP) and lamina propria (LP). The square area indicates a site of a previous transurethral resection (TUR) showing fibrosis of the bladder wall.

Standard radical cystectomy specimen includes the distal part of ureters, prostate, and seminal vesicles in men or urethra, adjacent vagina, and uterus in women. The organs adjacent to the bladder and the peritoneal lining allow to orientate the surgical specimen. Before dissection, it is recommended for cystectomy specimen an adequate fixation; this may be obtained either by distension of urinary bladder cavity with formalin injection (e.g., using a large gauge needle through the bladder dome or Foley catheter through the urethra) or by opening the bladder anteriorly from urethra to bladder dome before immersion in formalin (4). After adequate fixation, the orientated bladder specimen must be entirely and transversely sectioned at 5 mm intervals from bladder neck to dome, so that slices can be better compared with transverse tomographic scan or MRI (4, 8) (Figure 1A). The macroscopic description of the internal bladder surface should include the size, site, and appearance of tumor (papillary, solid, polypoid, or ulcerated) and the state of remaining mucosa. Moreover, the presence or absence of gross fat or serosa invasion should be recorded. When tumor is identified, the sampling should be adequate to its size (at least one section should be taken for each centimeter of tumor) and should allow to evaluate its deepest penetration on bladder wall, the grade, and the histological type. Sampling of normal appearing mucosa on different regions of the bladder wall should be made to detect occult multifocal carcinoma.

However, the tumor may not always be grossly visible, especially after re-TUR or pre surgical treatment, as a result of neoadjuvant therapy. In such cases, the sampling should be guided by prior surgical site, mucosal ulceration, or by cystoscopy or radiological images taken before tumor treatment. An extensive sampling is recommended in cystectomy with potential no residual tumor (4, 5, 8).

Whole mount technique can be used as an alternative to partial sampling by standard regular histological sections, even if whole mount method is not different to standard method in detecting adverse pathological features. Whole mount section advantages are a better view of bladder wall architecture and an easier comparison of the pathological findings with those obtained from radiological images (5) (Figure 1B).

## Pathology Reporting

The pathology report should include clinically relevant information as well as clinically useful gross and microscopic parameters. In this section, we consider the histological elements which should be present in the pathological report concerning both TUR/biopsy and cystectomy specimens. Currently, the International Collaboration on Cancer Reporting (ICCR) (19, 20) has elaborated a checklist for bladder cancer pathology report drafting considering dataset provided by several pathological anatomy organizations (http://www.iccr-cancer.org/datasets/published-datasets/urinary-male-genital/ut-biopsy-and-tr; and http://www.iccr-cancer.org/datasets/published-datasets/urinary-male-genital/bladder). In addition, ICCR has developed collaborations with other international cancer organizations responsible for neoplasm staging, such as American Joint Committee on Cancer (AJCC) and Union for international Cancer Control (UICC). The ICCR checklist includes the indications provided by the last WHO Classification of bladder tumor (21).

### Histological Tumor Types

**Figure 2:**
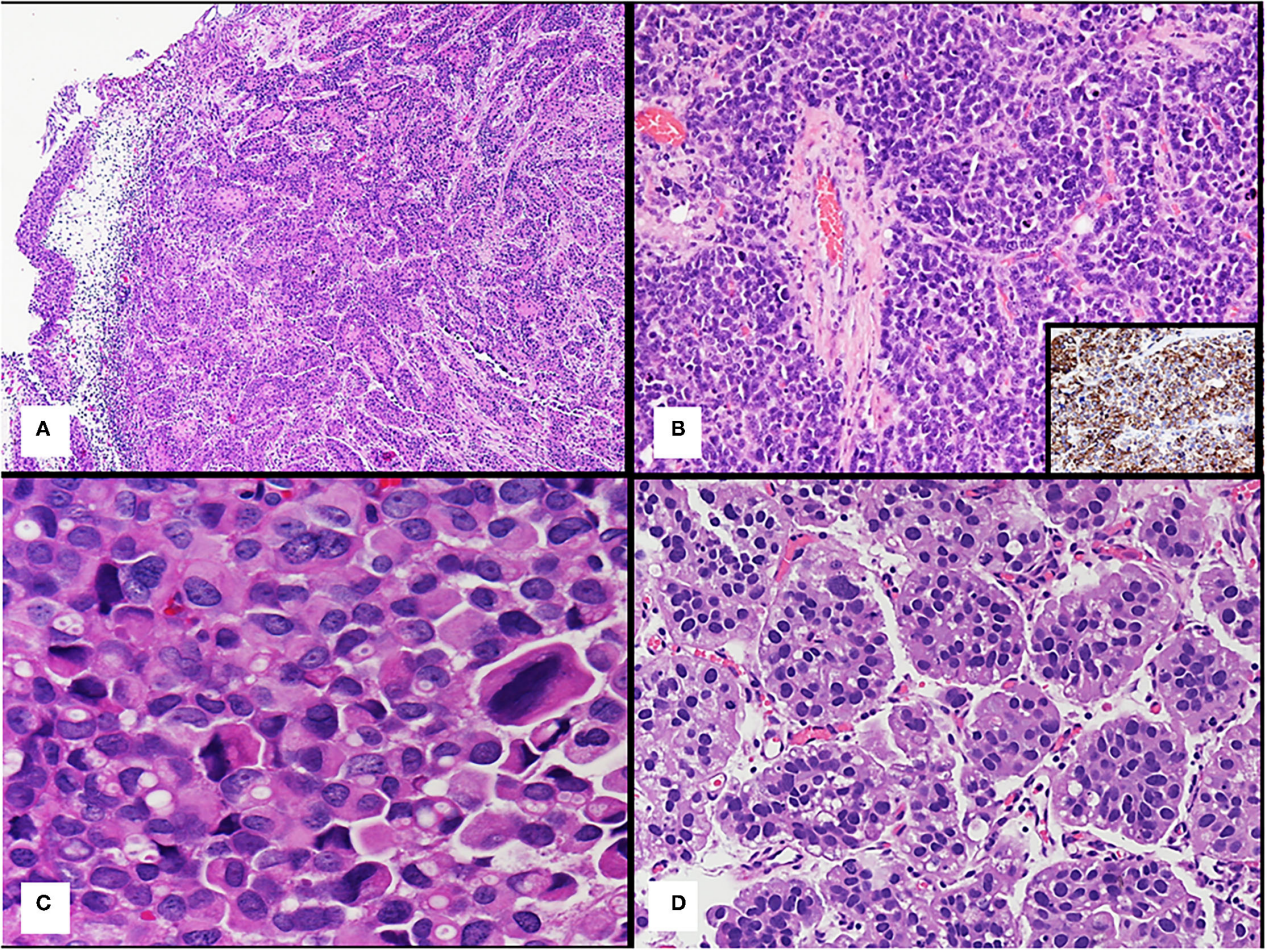
**(A)** Urothelial carcinoma with squamous divergent differentiation. **(B)** Bladder neuroendocrine tumor; the inset shows positive immunohistochemistry staining for synaptophysin of the tumor. **(C)** Plasmacytoid urothelial carcinoma. **(D)** Micropapillary urothelial carcinoma.

At present, different histological tumor types of bladder cancer are reported according to the 2016 WHO classification of urinary bladder tumors (21). The urothelial carcinoma is classified as such when there is any identifiable urothelial component, such as urothelial CIS. It is well-known that urothelial carcinoma may show unusual morphologic features that represent a divergent differentiation from 7 to 81% in various series (22–24). When urothelial carcinoma is not in pure form but shows divergent morphologies (Figure 2A), the histological tumor type retains the designation of urothelial carcinoma with associated histological subtype (e.g., squamous and glandular) and the percentage of each component of the tumor should be provided, because of its prognostic implication (22, 23, 25).

Neuroendocrine tumors (Figure 2B), such as small cell neuroendocrine carcinoma or large cell neuroendocrine carcinoma, are an exception to this rule, because regardless of the quantity of this component, it is recommended reporting all cases with a neuroendocrine carcinoma component as neuroendocrine tumor (19–21). These neuroendocrine tumors express immunohistochemical markers, such as synaptophysin (as shown inset in Figure 2B), chromogranin, and CD56 (21).

From a clinical point of view, the cases with a small cell neuroendocrine carcinoma component, are managed as small cell neuroendocrine carcinoma (26, 27). Few data exist about large cell neuroendocrine carcinomas, but they should probably be treated in the same way (28). Moreover, the ICCR suggested indicating neuroendocrine carcinoma component percentage because it influences carcinoma treatments, particularly the use of newest treatment, such as immunotherapy (19, 20).

WHO 2016 describes several variants of urothelial carcinoma and some of these have prognostic or therapeutic implications (21). These variants may represent a risk for bladder cancer under staging in the surgical specimens (29).

The nested type variant is a tumor with deceptively benign appearance that mimics von Brunn's nests and can be confused with von Brunn's nest hyperplasia if not invading the detrusor muscle. The tumor growth pattern varies from solid expansive to infiltrative nests without nuclear atypia that is observed most frequently in the deeper part of the tumor (30). Cytokeratins 20, 7, and p63 are expressed in nested type variant by immunohistochemistry (29, 31).

A nested type tumor is considered a high-grade carcinoma and when it has been compared with urothelial carcinoma, it has displayed more frequent advanced tumor stage and increased rate of nodal metastasis (32, 33), this may be related to morphological features of this variant (similar to von Brunn's nests) delaying diagnosis of malignancy (34). In any case, patients with nested variant compared with those with pure urothelial carcinoma at the same stage have similar oncological outcome with no difference in recurrence rate and survival when treated surgically (35).

Plasmacytoid/diffuse variant is characterized by individual cells that look like plasma cells and single cells with cytoplasmic vacuoles can be present (Figure 2C). This tumor shows no extracellular mucin production (29, 36) and displays a diffusely infiltrative growth pattern with minimal stromal reaction, and frequent peritoneal carcinomatosis. Plasmacytoid carcinoma typically express urothelial markers, such as p63 and GATA3 and CD138, a plasma-cell marker (29, 31).

At presentation, plasmacytoid variant has a greater chance for higher-stage disease when compared with conventional urothelial carcinoma, showing metastasis and surgical margin positivity. The positivity of margins is due to both the capacity of tumor cells spreading in single file and lack of desmoplastic reaction, that makes difficult to determine the surgical plane between the tumor and normal tissue.

Identification of plasmacytoid variant is important to ensure an adequate resection at the time of cystectomy (37).

Therefore, diagnosis of plasmacytoid variant is important in the first TUR specimens, because an immediate cystectomy should be considered in early invasive tumors (i.e., pT1) (38, 39) while advanced disease appears to be chemotherapy responsive (29).

Lymphoepithelioma-like carcinoma (LELC) variant of urothelial carcinoma resembles the nasopharynx lymphoepithelioma, but unlike this, it is not related to Epstein-Barr virus. It is composed of nests, sheets, and cord of poorly differentiated cells with pleomorphic nuclei, prominent nucleoli, and indistinct cytoplasmic borders with syncytial appearance. A characteristic feature of this tumor is a dense infiltrate of lymphoid cells that may mask the carcinoma cells (40).

Immunohistochemistry for cytokeratins (7 and 20) and urothelial markers (p63, GATA3, and uroplakin) highlights the epithelial cells for the diagnosis of LELC (29, 31).

Pure or predominant form of this variant appears to have a good prognosis with low metastatic potential (40) and a very favorable response to chemotherapy while mixed form LELC has a prognosis depending on the other variant present in the tumor. Therefore, cystectomy should be recommended in these last cases due to association with highest disease-free survival rate (8%) compared with TUR or partial cystectomy (41–43). A recent study showed that LELC tumors express PD-L1, this finding suggests to use immune checkpoint PD-L1 inhibitors as a therapeutic option (41).

Micropapillary urothelial carcinoma is characterized by small clusters of tumor cells without fibrovascular cores surrounded by empty spaces due to prominent retraction artifact that may mimic vascular invasion (44) (Figure 2D).

This variant shows positive stain for cytokeratin 7 and 20, epithelial membrane antigen (EMA) and Mucin 1 (MUC1) (29, 31).

A micropapillary tumor is an aggressive variant of urothelial carcinoma, at the time of detection more than 95% of these tumors are muscle invasive and in advanced stage, and lymph node involvement occur up to 35% of the patients (45, 46). This variant is frequently mixed with conventional urothelial carcinoma or other variant, and some studies suggest that any amount of micropapillary variant, even <10% is significant in urothelial carcinoma and should be reported (45, 47). In addition, some studies show contradictory results concerning micropapillary variant aggressiveness compared with pure urothelial carcinoma in the patients who underwent cystectomy (48).

In any case, non-muscle invasive micropapillary tumor is associated with high rate of progression to muscle invasive disease, and some studies have observed that this tumor is unresponsive to intravesical therapy with BCG so early cystectomy is considered the standard management in most urological centers (29, 48, 49).

Considering muscle-invasive disease, protocols for neoadjuvant chemotherapy administration are not clear, so some authors indicated the immediate cystectomy while others recommend cystectomy with neoadjuvant chemotherapy (41, 50).

The molecular studies have shown that micropapillary carcinoma is characterized by HER2Neu overexpression and activation of miR-296 and RUVBL1 target genes showing relevant insights for future targeting therapy (47, 51).

Sarcomatoid urothelial carcinoma is an aggressive variant of urothelial carcinoma characterized by both epithelial and mesenchymal malignant differentiation, and undifferentiated high-grade spindle cell sarcoma is the mesenchymal component observed most frequently. Heterologous malignant elements may be present (e.g., osteosarcoma, chondrosarcoma, rhabdomyosarcoma, and leiomyosarcoma) (52, 53). In sarcomatoid urothelial carcinoma, the two components, carcinomatous and sarcomatous, are present in variable amount, but in the most cases sarcomatous component represents >50%. This variant may show prominent myxoid and sclerosing stroma, and that makes the diagnosis challenging.

This malignant neoplasm can be confused with spindle cell benign neoplasm or it can be under staging, especially in the TUR specimens, because spindled morphology of this neoplasia may obscure the muscularis (2, 29).

As previously described, sarcomatoid urothelial carcinoma is a biphasic tumor and immunohistochemical features evidence this aspect. The carcinomatous component is positive for the epithelial markers (i.e., AE1/AE3 and keratin CAM 5.2) and for EMA, as well as it is positive for mesenchymal marker as vimentin in ~80–90% of tumors. In addition, the sarcomatous component is always positive for vimentin while it can express one or more epithelial markers. High molecular-weight cytokeratin is the marker most frequently expressed in the sarcomatous component. Moreover, immunohistochemical expression of urothelial differentiation markers, such as p63 and GATA3, although focal, can be useful for the diagnosis of this variant (21, 29, 31).

Sarcomatoid urothelial carcinoma frequently occurs at an advanced stage and it has a poor prognosis when compared with pure urothelial carcinoma (52, 53).

The survival for this type of carcinoma does not appear different in cases underwent to cystectomy compared with those receiving neoadjuvant or adjuvant chemotherapy (54).

### Histological Tumor Grade

Histological tumor grade is a crucial parameter especially for non-invasive papillary urothelial tumor to guide the choice of therapy. The 2016 WHO (21) and more recently the ICCR (19, 20) recommend to use the same grade system adopted by WHO 2004 based on those initially proposed by the International Society of Urological Pathology (ISUP) in 1997 (55), whereas the use of other grading systems is considered as optional and it should be indicated.

The 2016 WHO classification system includes two categories of non-invasive bladder tumor, i.e., flat and papillary. The first is named urothelial CIS and the second type consist of papillary urothelial neoplasm of low malignant potential (PUNLMP) and papillary urothelial carcinoma.

A urothelial CIS is a flat non-invasive urothelial lesion of variable thickness, devoid of papillary structures containing cytologically malignant cells. It is very often multifocal and isolated ~3% of cases. It is present with a synchronous non-muscle invasive urothelial carcinoma or with muscle invasive carcinoma in 50 and 60%, respectively (21, 56).

Papillary urothelial neoplasm of low malignant potential is considered a neoplasm unable to invade or metastasize, whereas papillary urothelial carcinoma is divided in two-tiered group: low and high-grade reflecting the different risk of progression to invasive carcinoma and death from bladder cancer (57). It is well-known that papillary urothelial carcinoma can present grade heterogeneity that has been reported in 3–43% of papillary urothelial lesions. Some studies indicated that mixed grade tumors should be labeled as high-grade tumors considering the percentage of high-grade components, but because of limited data, the cut-off utilized seem to be arbitrary (58–60). A recent study has demonstrated that low-grade areas in mixed grade papillary urothelial cancer showed molecular changes associated with disease progression (e.g., CDKN2A deletion) suggesting that molecular changes occur early and before morphological changes (61).

These findings support the current recommendation by the WHO 2016 and ICCR that the grade of the tumor depends on the highest-grade present in the lesion, so even if the lesion shows focal or minimal high-grade component, it has to be considered a high-grade tumor. In addition, the International Consultation of Urological Disease (ICUD) suggests that the percentage of the tumor high grade component should be recorded if it is <10% in the pathological report (62). Regarding invasive urothelial carcinoma, it should be considered as high grade (19, 62).

### Extent of Invasion

**Figure 3:**
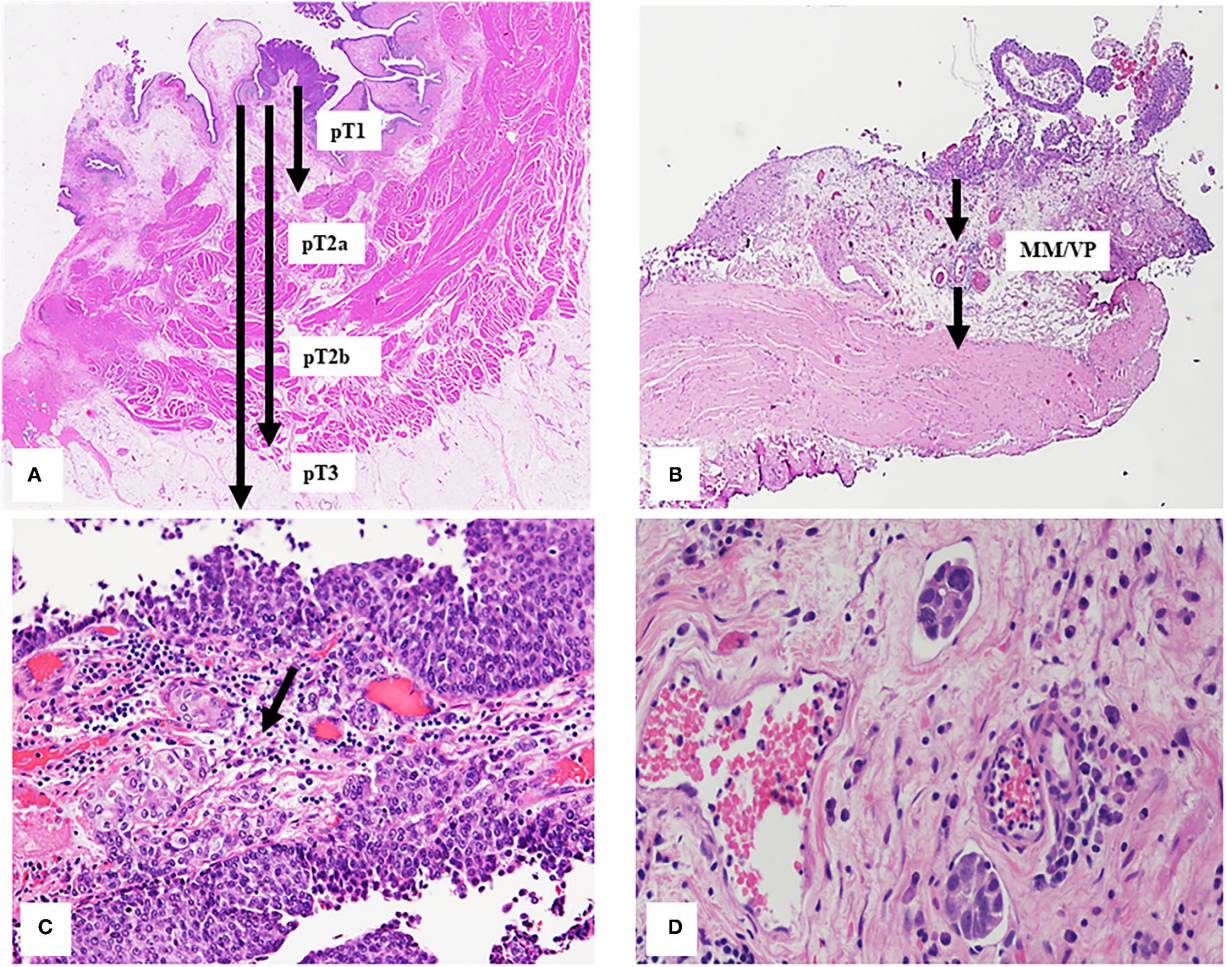
**(A)** Extension of bladder tumor transmurally invasion classified considering 2017 version of AJCC TNM classification as described in the text. The arrows indicate different depths in relation to the different histological tumor stages. **(B)** Bladder wall Black arrows indicate the vascular plexus (VP) and the muscularis mucosae (MM), these divide the lamina propria in two part and represent the landmark to subclassified T1 tumors. **(C)** The arrow indicates the urothelial carcinoma infiltrating the lamina propria. **(D)** Small groups of tumor cells into two lymphatic vessels.

Tumor invasion extension through the bladder wall is the criteria to assign the pathologic stage (pT) and at present, the 2017 version of AJCC Tumor-Nodes-Metastasis (TNM) classification is used (63, 64) (Figure 3A). Tumor staging can be difficult for pathologist so in this section, we will discuss the most common problems related to bladder cancer staging in different conditions of surgical specimen.

### TUR or Biopsy

#### Pathologic Stage 1 (pT1)

Pathologic stage 1 (pT1) is defined by presence of tumor cells invading the sub-epithelial connective tissue (lamina propria), underneath the basement membrane, but not the muscularis propria (MP).

Some common diagnostic pitfalls are bound to surgical and excision factors, such as cautery injury, tangential section, or poor orientation of specimens. Other difficulty is due to the tissue reaction factors, such as desmoplastic stroma, inflammatory infiltrate, which may obscure single tumor cell infiltrating the lamina propria, or bladder epithelium and wall iatrogenic changes due to radiation therapy for pelvic cancers. In addition, tumor characteristics can make difficult the interpretation of the staging, such as some urothelial carcinoma variants (e.g., nested type or micropapillary) or CIS spread into von Brunn's nest (8, 65, 66) as well as the involvement of muscle fibers by invasive tumor. Several studies showed tumor up-staging (from 3 to 13%) or down-staging (from 15 to 56%) for pT1cases (67, 68) and also between expert genitourinary pathologists, a full agreement was reached in 47% of the pT1 cases (68) because the diagnosis of lamina propria invasion or the infiltration of the muscularis propria can be challenging (8, 66). In fact, the muscularis mucosae (MM) contained in the lamina propria can represent a confounding element to a correct diagnosis because the smooth muscles fibers constitute both MM and MP. In addition, the MM is not a complete layer in bladder and can be hyperplastic especially in the dome and the lamina propria is thinner in the bladder trigone and neck regions where, on the contrary, the MP is both thicker and more superficial (65, 66). Recently, smoothelin was proposed as a promising immunohistochemical marker to distinguish MP from MM which is usually weak or negative for smoothelin. It can be difficult to discriminate MM and MP in the TUR specimens when smoothelin staining is modest and without MP present as internal reference. Due to these limitations, the use of smoothelin is not currently recommended routinely (69). Other muscle markers, such as desmin and caldesmon were tested but unfortunately do not show unequal staining of MM and MP. If indecision between MM or MP involvement remains, this should be commented in the report (20). Last WHO Classification, CAP, ICUD, and ICCR recommend substaging of pT1 but no indication is provided on methods to use for evaluating invasion extension. The anatomical or quantitative methods were proposed in literature to sub classify pT1 tumor, most of them correlate with recurrence free survival, progression free survival, and cancer specific survival. The anatomical method to sub classify pT1 tumor is based on the deep of invasion using MM or LP vascular plexus (its surrogate) as the histological landmark (Figure 3B). So, pT1 tumors can be classified in two or three sub classes (pT1a, b, or c) (70, 71). Recent meta-analysis showed that clinicians should treat patients with T1b/c substaging as having risk on a par with invasive bladder cancer (72).

The most frequently used quantitative methods to substage pT1 tumors is measuring the depth or maximum linear length of the invasive focus. The depth of invasive tumor is taken perpendicular to the mucosal surface while the maximum linear length of the invasive tumor can be the aggregated length of invasion foci; this last method is less affected by the orientation of the specimen (73–75) (Figure 3C). Over time, different cut-off points have been proposed for both methods to provide more information concerning risk of pT1 tumor recurrence and progression. Cheng et al. (76) have suggested a cut-off of 1.5 mm of depth of invasion calculated from basement membrane, others have suggested different aggregated invasive tumor lengths (73–75, 77, 78). In particular, Hu et al. (78) suggested 5 mm as cut-off of aggregated invasive tumor length for pT1 tumor recurrence, while Leivo et al. (74) proposed 2.3 mm as the optimal cut-off, which is larger than previously tested cut-offs of ~0.5 and 1.0 mm measurements (73, 75) but smaller than more generous of 5 mm proposed by Hu et al. (78) for risk of pT1 tumor progression.

Regardless of the method used, an assessment of the depth and/ or extent of lamina propria invasion in pT1 cases should be provided in the pathological report.

### Cystectomy

#### Pathologic Stage 0 (pT0)

Pathologic stage 0 (pT0) is assigned when residual tumor is not present in the cystectomy specimens after a previous cancer diagnosis in biopsy or TUR specimens or after neoadjuvant chemotherapy (yT0) (63, 64). The rate of pT0 is from 5 to 20% in contemporary cystectomy series without preoperative chemotherapy (79, 80) and comes up to 46% in cystectomy series after neoadjuvant chemotherapy (81).

Clinical indications about the site of the neoplasia should be considered when cystectomy specimens are evaluated for residual disease if no grossly apparent lesion exist or to find the site of previous TUR. In both cases, the suspicious area should be completely evaluated and if no residual cancer is found the case can be reported as pT0 (19).

#### Pathologic Stage 2 (pT2)

Pathologic stage 2 (pT2) bladder carcinoma is defined by tumor invasion into muscularis propria. pT2 bladder cancer is sub classified in two categories on the base of depth invasion of the muscularis propria: in pT2a the tumor invades the inner half (superficial part), while in pT2b the tumor invades the outer half (deep part) of the muscle wall (63, 64). Detrusor muscle anatomy does not always allow an easy distinction between inner and outer part of muscularis propria, and pathologist has to divide arbitrarily the muscle wall (65). These factors caused contradictory results in studies investigating oncological outcome of pT2 substaging in radical cystectomy (82–84).

#### Pathologic Stage 3 (pT3)

Pathologic stage (pT3) bladder carcinoma is defined by tumor invasion into peri-vesical soft tissue, such as peri-vesical fat. Fat invasion evaluation could seem easy but it can be challenging because the interpretation of microscopic peri-vesical tissue invasion could be subjective (19) as the junction between the outer layer of the muscularis propria and the peri-vesical fat is badly defined. The deeper part of muscularis propria shows haphazardly separated muscle bundles without clear demarcation with adipose peri-vesical tissue. Anatomical aspects and tumor related factors as dense fibrosis, desmoplasia, obscuring inflammation, and lympho-vascular invasion should be considered in the interpretation of tumor invasion beyond the muscularis propria (85). Invasive carcinoma surrounded by desmoplastic reaction, even if it does not touch the peri vesical fat but it is beyond the muscularis propria, should be considered pT3 as recommended by ICCR (19). pT3 bladder cancer is sub classified in two categories: pT3a (tumor with microscopic extravesical extension) and pT3b (tumor with gross extravesical extension). A tumor described as grossly involving the peri-vesical soft tissue requires histologic confirmation before it is considered in pT3b category (19).

### Lymph Vascular Invasion

Lymph vascular invasion (LVI) is characterized by the presence of small group of tumor cells into lymphatic or blood vessels (Figure 3D). Its identification can be misleading in surgical specimens because retraction artifact around nest of invasive tumor cells (e.g., micropapillary variant) or peritumoral stroma retraction (86).

LVI detection can be difficult using hematoxylin and eosin-stained section, so immunohistochemistry technique (CD31, CD34, and D240) can be necessary but conflicting data exist on immunohistochemical staining for diagnosis of LVI in bladder cancer, so this technique should be used only in selected equivocal cases (1). Several studies suggested that LVI is an independent predictor of poor disease outcome both in TUR and cystectomy cases (1, 87). LVI presence should be indicated in the pathological report as the ICCR requested.

## Conclusion

In conclusion, we can affirm that the handling and pathological evaluation of bladder cancer surgical specimens are crucial to provide guidance for patient treatment. In this review, we have addressed the most discussed topics for the interpretation of surgical bladder samples requested from the major international pathology organizations that should be related in the pathological report. In particular, we considered the major issues that arises in the evaluation of both TUR and cystectomy samples to improve the collaboration between pathologists and urologists.

## Author Contributions

RMa contributed to the conception of the manuscript. RMa and DM contributed to the writing of the manuscript. GT, LP, and RMo contributed to the critical revision and final approval of the article. All authors contributed to the article and approved the submitted version.

## Conflict of Interest

The authors declare that the research was conducted in the absence of any commercial or financial relationships that could be construed as a potential conflict of interest.

## Publisher's Note

All claims expressed in this article are solely those of the authors and do not necessarily represent those of their affiliated organizations, or those of the publisher, the editors and the reviewers. Any product that may be evaluated in this article, or claim that may be made by its manufacturer, is not guaranteed or endorsed by the publisher.

## References

[1] Amin MB McKenney JK Paner GP Hansel DE Grignon DJ Montironi R. ICUD-EAU international consultation on bladder cancer 2012: pathology. Eur Urol. (2013) 63:16–35. 10.1016/j.eururo.2012.09.063 23083804

[2] Hansel DE Amin MB Comperat E Cote RJ Knüchel R Montironi R. A contemporary update on pathology standards for bladder cancer: transurethral resection and radical cystectomy specimens. Eur Urol. (2013) 63:321–32. 10.1016/j.eururo.2012.10.008 23088996

[3] Hansel DE Miller JS Cookson MS Chang SS. Challenges in the pathology of non-muscle-invasive bladder cancer: a dialogue between the urologic surgeon and the pathologist. Urology. (2013) 81:1123–30. 10.1016/j.urology.2013.01.027 23522296 PMC5224832

[4] Chandra A Griffiths D McWilliam LJ. Best practice: gross examination and sampling of surgical specimens from the urinary bladder. J Clin Pathol. (2010) 63:475–9. 10.1136/jcp.2009.071191 20498023

[5] Mazzucchelli R Scarpelli M Lopez-Beltran A Cheng L Di Primio R Montironi R. A contemporary update on pathology reporting for urinary bladder cancer. Int J Immunopathol Pharmacol. (2012) 25:565–71. 10.1177/039463201202500302 23058006

[6] Compérat E Varinot J Moroch J Eymerit-Morin C Brimo F. A practical guide to bladder cancer pathology. Nat Rev Urol. (2018) 15:143–54. 10.1038/nrurol.2018.2 29384523

[7] Lopez-Beltran A Luque RJ Mazzucchelli R Scarpelli M Montironi R. Changes produced in the urothelium by traditional and newer therapeutic procedures for bladder cancer. J Clin Pathol. (2002) 55:641–7. 10.1136/jcp.55.9.641 12194991 PMC1769754

[8] Cheng L Montironi R Davidson DD Lopez-Beltran A. Staging and reporting of urothelial carcinoma of the urinary bladder. Mod Pathol. (2009) 22:S70–95. 10.1038/modpathol.2009.1 19494855

[9] Beaghler M Grasso M III. Flexible cystoscopic bladder biopsies: a technique for outpatient evaluation of the lower urinary tract urothelium. Urology. (1994) 44:756–9. 10.1016/S0090-4295(94)80223-8 7974952

[10] van der Meijden A Oosterlinck W Brausi M Kurth KH Sylvester R de Balincourt C. Significance of bladder biopsies in Ta,T1 bladder tumors: a report from the EORTC genito-urinary tract cancer cooperative group. EORTC-GU group superficial bladder committee. Eur Urol. (1999) 35:267–71. 10.1159/000019859 10419345

[11] Babjuk M Burger M Compérat EM Gontero P Mostafid AH Palou J. European association of urology guidelines on non-muscle-invasive bladder cancer (TaT1 and carcinoma *in situ*) - 2019 update. Eur Urol. (2019) 76:639–57. 10.1016/j.eururo.2019.08.016 31443960

[12] Brausi M Witjes JA Lamm D Persad R Palou J Colombel M. A review of current guidelines and best practice recommendations for the management of nonmuscle invasive bladder cancer by the international bladder cancer group. J Urol. (2011) 186:2158–67. 10.1016/j.juro.2011.07.076 22014799

[13] Territo A Bevilacqua G Meneghetti I Mercadé A Breda A. En bloc resection of bladder tumors: indications, techniques, and future directions. Curr Opin Urol. (2020) 30:421–7. 10.1097/MOU.0000000000000737 32205806

[14] D'Souza N Verma A. Holmium laser transurethral resection of bladder tumor: our experience. Urol Ann. (2016) 8:439–43. 10.4103/0974-7796.190815 28057988 PMC5100149

[15] Hu J. En bloc transurethral resection with hybrid knife for treatment primary non-muscle-invasive bladder cancer: a single-center, controlled trial based on pathological staging. J Urol. (2018) 199:e615. 10.1016/j.juro.2018.02.1481

[16] Teoh JYC MacLennan S Wai-Shun Chan V Miki J Lee HY Chiong E. An international collaborative consensus statement on en bloc resection of bladder tumour incorporating two systematic reviews, a two-round delphi survey, and a consensus meeting. Eur Urol. (2020) 78:546–69. 10.1016/j.eururo.2020.04.059 32389447

[17] Yang Y Liu C Yan X Li J Yang X. En bloc tumor resection, optical molecular imaging, and the potential synergy of the combination of the two techniques in bladder cancer. Front Oncol. (2021) 11:638083. 10.3389/fonc.2021.638083 33796465 PMC8008058

[18] Richterstetter M Wullich B Amann K Haeberle L Engehausen DG Goebell PJ. The value of extended transurethral resection of bladder tumour (TURBT) in the treatment of bladder cancer. BJU Int. (2012) 110:E76–9. 10.1111/j.1464-410X.2011.10904.x 22313727

[19] Compérat E Srigley JR Brimo F Delahunt B Koch M Lopez-Beltran A. Dataset for the reporting of carcinoma of the bladder-cystectomy, cystoprostatectomy and diverticulectomy specimens: recommendations from the international collaboration on cancer reporting (ICCR). Virchows Arch. (2020) 476:521–34. 10.1007/s00428-019-02727-1 31915958

[20] Varma M Srigley JR Brimo F Compérat E Delahunt B Koch M. Dataset for the reporting of urinary tract carcinoma-biopsy and transurethral resection specimen: recommendations from the international collaboration on cancer reporting (ICCR). Mod Pathol. (2020) 33:700–12. 10.1038/s41379-019-0403-9 31685965

[21] Moch H Humphrey PA Ulbright TM Reuter RE. WHO Classification of Tumours of the Urinary System and Male Genital Organs. Lyon: IARC Press (2016) 78p. 10.1016/j.eururo.2016.02.029 26996659

[22] Amin MB. Histologic variants of urothelial carcinoma: diagnostic, therapeutic and prognostic implications. Modem Pathol. (2009) 22:S96–118. 10.1038/modpathol.2009.26 19494856

[23] Billis A Schenka AA Ramos CC Carneiro LT Araujo V. Squamous and/or glandular differentiation in urothelial carcinoma: prevalence and significance in transurethral resections of the bladder. Int Urol Nephro1. (200l) 33:631–3. 10.1023/A:1020597611645 12452615

[24] Chalasani V Chin JL Izawa Jl. Histologic variants of urothelial bladder cancer and non urothelial histology in bladder cancer. Can Urol Assoc J. (2009) 3:Sl93–8. 10.5489/cuaj.1195 20019984 PMC2792446

[25] Wasco MJ Daignault S Zhang Y Kunju LP Kinnaman M Braun T. Urothelial carcinoma with divergent histologic differentiation (mixed histologic features) predicts the presence of locally advanced bladder cancer when detected at transurethral resection. Urolog. (2007) 70:69–74. 10.1016/j.urology.2007.03.033 17656211

[26] Alanee S Alvarado-Cabrero I Murugan P Kumar R Nepple KG Paner GP. Update of the international consultation on urological diseases on bladder cancer 2018: non-urothelial cancers of the urinary bladder. World J Urol. (2019) 37:107–14. 10.1007/s00345-018-2421-5 30069580

[27] Warrick JI Sjödahl G Kaag M Raman JD Merrill S Shuman L. Intratumoral heterogeneity of bladder cancer by molecular subtypes and histologic variants. Eur Urol. (2019) 75:18–22. 10.1016/j.eururo.2018.09.003 30266310

[28] Zhou HH Liu LY Yu GH Qu GM Gong PY Yu X. Analysis of clinicopathological features and prognostic factors in 39 cases of bladder neuroendocrine carcinoma. Anticancer Res. (2017) 37:4529–37. 10.21873/anticanres.11850 28739749

[29] Lopez-Beltran A Henriques V Montironi R Cimadamore A Raspollini MR Cheng L. Variants and new entities of bladder cancer. Histopathology. (2019) 74:77–96. 10.1111/his.13752 30565299

[30] Lopez-Beltran A Cheng L. Histologic variants of urothelial carcinoma: differential diagnosis and clinical implications. Hum. Pathol. (2006) 37:1371–88. 10.1016/j.humpath.2006.05.009 16949919

[31] Moschini M D'Andrea D Korn S Irmak Y Soria F Compérat E. Characteristics and clinical significance of histological variants of bladder cancer. Nat Rev Urol. (2017) 14:651–68. 10.1038/nrurol.2017.125 28895563

[32] Lopez Beltran A Cheng L Montironi R Blanca A Leva M Rouprêt M. Clinicopathological characteristics and outcome of nested carcinoma of the urinary bladder. Virchows Arch. (2014) 465:199–205. 10.1007/s00428-014-1601-y 24878757

[33] Wasco MJ Daignault S Bradley D Shah RB. Nested variant of urothelial carcinoma: a clinicopathologic and immunohistochemical study of 30 pure and mixed cases. Hum Pathol. (2010) 41:163–71. 10.1016/j.humpath.2009.07.015 19800100

[34] Mally AD Tin AL Lee JK Satasivam P Cha EK Donat SM. Clinical outcomes of patients with T1 nested variant of urothelial carcinoma compared to pure urothelial carcinoma of the bladder. Clin Genitourin Cancer. (2017) 17:30199–204. 10.1016/j.clgc.2017.07.002 28802887 PMC5767538

[35] Linder BJ Frank I Cheville JC Thompson H Thapa P Tarrell RF. Outcomes following radical cystectomy for nested variant of urothelial carcinoma: a matched cohort analysis. J Urol. (2013) 189:1670–5. 10.1016/j.juro.2012.11.006 23142686

[36] Keck B Stoehr R Wach S Rogler A Hofstaedter F Lehmann J. The plasmacytoid carcinoma of the bladder – rare variant of aggressive urothelial carcinoma. Int J Cancer. (2011) 15:346–54. 10.1002/ijc.25700 20878954

[37] Cockerill PA Cheville JC Boorjian SA Blackburne A Thapa P Tarrell RF. Outcomes following radical cystectomy for plasmacytoid urothelial carcinoma: defining the need for improved local cancer control. Urology. (2017) 102:143–7. 10.1016/j.urology.2016.09.053 27865750

[38] Kim DK Kim JW Ro JY Lee HS Park JY Ahn HK. Plasmacytoid variant urothelial carcinoma of the bladder: a systematic review and meta-analysis of clinicopathological features and survival outcomes. J Urol. (2020) 204:215–23. 10.1097/JU.0000000000000794 32003614

[39] Sood S Paner GP. Plasmacytoid urothelial carcinoma: an unusual variant that warrants aggressive management and critical distinction on transurethral resections. Arch Pathol Lab Med. (2019) 143:1562–7. 10.5858/arpa.2018-0139-RS 30865491

[40] Lopez-Beltran A Luque RJ Vicioso L Anglada F Requena MJ Quintero A. Lymphoepithelioma-like carcinoma of the urinary bladder: a clinicopathologic study of 13 cases. Virchows Arch. (2001) 438:552–7. 10.1007/s004280000378 11469686

[41] Lobo N Shariat SF Guo CC Fernandez MI Kassouf W Choudhury A. What is the significance of variant histology in urothelial carcinoma? Eur Urol Focus. (2020) 15:653–63. 10.1016/j.euf.2019.09.003 31530497

[42] Williamson SR Zhang S Lopez-Beltran A Shah RB Montironi R Tan PH. Lymphoepithelioma- like carcinoma of the urinary bladder: clinicopathologic, immunohistochemical, and molecular features. Am J Surg Pathol. (2011) 35:474–83. 10.1097/PAS.0b013e31820f709e 21383609

[43] Yang AW Pooli A Lele SM Kim IW Davies JD LaGrange CA. Lymphoepithelioma-like, a variant of urothelial carcinoma of the urinary bladder: a case report and systematic review for optimal treatment modality for disease-free survival. BMC Urol. (2017) 17:34. 10.1186/s12894-017-0224-4 28449665 PMC5408364

[44] Amin MB Ro JY El-Sharkawy T Lee KM Troncoso P Silva EG. Micropapillary variant of transitional cell carcinoma of the urinary bladder histologic pattern resembling ovarian papillary serous carcinoma. Am J Surg Pathol. (1994) 18:1224–32. 10.1097/00000478-199412000-00005 7977945

[45] Compérat E Roupret M Yaxley J Reynolds J Varinot J Ouzaïd I. Micropapillary urothelial carcinoma of the urinary bladder: a clinicopathological analysis of 72 cases. Pathology. (2010) 42:650–4. 10.3109/00313025.2010.522173 21080874

[46] Kamat AM Dinney CPN Gee JR Grossman HB Siefker-Radtke AO Tamboli P. Micropapillary bladder cancer: a review of the University of Texas MD Anderson cancer center experience with 100 consecutive patients. Cancer. (2007) 110:62–7. 10.1002/cncr.22756 17542024

[47] Lopez-Beltran A Montironi R Blanca A Cheng L. Invasive micropapillary urothelial carcinoma of the bladder. Hum Pathol. (2010) 4:1159–64. 10.1016/j.humpath.2009.11.018 20381120

[48] Abufaraj M Foerster B Schernhammer E Moschini M Kimura S Hassler MR. Micropapillary urothelial carcinoma of the bladder: a systematic review and meta-analysis of disease characteristics and treatment outcomes. Eur Urol. (2019) 75:649–58. 10.1016/j.eururo.2018.11.052 30553613

[49] Willis DL Fernandez MI Dickstein RJ Parikh S Shah JB Pisters LL. Clinical outcomes of cT1 micropapillary bladder cancer. J Urol. (2015) 193:1129–34. 10.1016/j.juro.2014.09.092 25254936 PMC4687395

[50] Veskimäe E Espinos EL Bruins HM Yuan Y Sylvester R Kamat AM. What is the prognostic and clinical importance of urothelial and nonurothelial histological variants of bladder cancer in predicting oncological outcomes in patients with muscle-invasive and metastatic bladder cancer? A European association of urology muscle invasive and metastatic bladder cancer guidelines panel systematic review. Eur Urol. (2019) 2:625–42. 10.1016/j.euo.2019.09.003 31601522

[51] Guo CC Dadhania V Zhang L Majewski T Bondaruk J Sykulski M. Gene expression profile of the clinically aggressive micropapillary variant of bladder cancer. Eur Urol. (2016) 70:611–20. 10.1016/j.eururo.2016.02.056 26988609 PMC5804336

[52] Cheng L Zhang S Alexander R Maclennan GT Hodges KB Harrison BT. Sarcomatoid carcinoma of the urinary bladder: the final common pathway of urothelial carcinoma dedifferentiation. Am J Surg Pathol. (2011) 35:e34–46. 10.1097/PAS.0b013e3182159dec 21490442

[53] Sanfrancesco J McKenney JK Leivo MZ Gupta S Elson P Hansel DE. Sarcomatoid urothelial carcinoma of the bladder: analysis of 28 cases with emphasis on clinicopathologic features and markers of epithelial–mesenchymal transition. Arch Pathol Lab Med. (2016) 140:543–1. 10.5858/arpa.2015-0085-OA 27031776

[54] Berg S D'Andrea D Vetterlein MW Cole AP Fletcher SA Krimphove MJ. Impact of adjuvant chemo- therapy in patients with adverse features and variant histology at radical cystectomy for muscle-invasive carcinoma of the bladder: does histologic subtype matter? Cancer. (2019) 125:1449–58. 10.1002/cncr.31952 30620387

[55] Epstein JI Amin MB Reuter VR Mostofi FK. The world health organization/international society of urological pathology consensus classification of urothelial (transitional cell) neoplasms of the urinary bladder. Bladder consensus conference committee. Am J Surg Pathol. (1998) 22:1435–48. 10.1097/00000478-199812000-00001 9850170

[56] Akhtar M Al-Bozom IA Ben Gashir M Taha NM Rashid S Al-Nabet ADMH. Urothelial carcinoma *in situ* (CIS): new insights. Adv Anat Pathol. (2019) 26:313–19. 10.1097/PAP.0000000000000239 31149909

[57] Pan CC Chang YH Chen KK Yu HJ Sun CH Ho DM. Prognostic significance of the 2004 WHO/ISUP classification for prediction of recurrence, progression, and cancer-specific mortality of non-muscle-invasive urothelial tumors of the urinary bladder: a clinicopathologic study of 1,515 cases. Am J Clin Pathol. (2010) 133:788–95. 10.1309/AJCP12MRVVHTCKEJ 20395527

[58] Cheng L Neumann RM Nehra A Spotts BE Weaver AL Bostwick DG. Cancer heterogeneity and its biologic implications in the grading of urothelial carcinoma. Cancer. (2000) 88:1663–70. 10.1002/(SICI)1097-0142(20000401)88:7<1663::AID-CNCR21>3.0.CO;2-8 10738225

[59] Gofrit ON Pizov G Shapiro A Duvdevani M Yutkin V Landau EH. Mixed high and low grade bladder tumors — are they clinically high or low grade? J Urol. (2014) 191:1693–6. 10.1016/j.juro.2013.11.056 24316096

[60] Reis LO Taheri D Chaux A Guner G Mendoza Rodriguez MA Bivalacqua TJ. Significance of a minor high-grade component in a low-grade noninvasive papillary urothelial carcinoma of bladder. Hum Pathol. (2016) 47:20–5. 10.1016/j.humpath.2015.09.007 26520419

[61] Downes MR Weening B van Rhijn BW Have CL Treurniet KM van der Kwast TH. Analysis of papillary urothelial carcinomas of the bladder with grade heterogeneity: supportive evidence for an early role of CDKN2A deletions in the FGFR3 pathway. Histopathology. (2017) 70:281–9. 10.1111/his.13063 27530957

[62] Compérat E Babjuk M Algaba F Amin M Brimo F Grignon D. SIU-ICUD on bladder cancer: pathology. World J Urol. (2019) 37:41–50. 10.1007/s00345-018-2466-5 30218308

[63] Bochner BH Hensel DE Efstathiou JA Konety B Lee CT McKiernan JM. Urinary bladder. In: Amin MB, editor. AJCC Cancer staging manual. Chicago: Springer (2017). p. 757.

[64] Lopez-Beltran A Cheng L. Stage T1 bladder cancer diagnostic criteria and pitfalls. Pathology. (2021) 53:67–85. 10.1016/j.pathol.2020.09.014 33153725

[65] Paner GP Montironi R Amin MB. Challenges in pathologic staging of bladder cancer: proposals for fresh approaches of assessing pathologic stage in light of recent studies and observations pertaining to bladder histoanatomic variances. Adv Anat Pathol. (2017) 24:113–27. 10.1097/PAP.0000000000000152 28398951

[66] Raspollini MR Montironi R Mazzucchelli R Cimadamore A Cheng L Lopez-Beltran A. pT1 high-grade bladder cancer: histologic criteria, pitfalls in the assessment of invasion, and substaging. Virchows Arch. (2020) 477:3–16. 10.1007/s00428-020-02808-6 32296929

[67] Bol MG Baak JP Buhr-Wildhagen S Kruse AJ Kjellevold KH Janssen EA. Reproducibility and prognostic variability of grade and lamina propria invasion in stages Ta, T1 urothelial carcinoma of the bladder. J Urol. (2003) 169:1291–4. 10.1097/01.ju.0000055471.78783.ae 12629345

[68] Compérat E Egevad L Lopez-Beltran A Camparo P Algaba F Amin M. An interobserver reproducibility study on invasiveness of bladder cancer using virtual microscopy and heatmaps. Histopathology. (2013) 63:756–66. 10.1111/his.12214 24102813

[69] Amin MB Trpkov K Lopez-Beltran A Grignon D Members Members of the ISUP Immunohistochemistry in Diagnostic Urologic Pathology Group. Best practices recommendations in the application of immunohistochemistry in the bladder lesions: report from the international society of urologic pathology consensus conference. Am J Surg Pathol. (2014) 38:e20–34. 10.1097/PAS.0000000000000240 25029121

[70] Orsola A Trias I Raventós CX Español I Cecchini L Búcar S. Initial high-grade T1 urothelial cell carcinoma: feasibility and prognostic significance of lamina propria invasion microstaging (T1a/b/c) in BCG-treated and BCG-non-treated patients. Eur Urol. (2005) 48:231–8. 10.1016/j.eururo.2005.04.013 15963635

[71] Rouprêt M Seisen T Compérat E Larré S Mazerolles C Gobet F. Prognostic interest in discriminating muscularis mucosa invasion (T1a vs T1b) in nonmuscle invasive bladder carcinoma: French national multicenter study with central pathology review. J Urol. (2013) 189:2069–76. 10.1016/j.juro.2012.11.120 23201497

[72] Martin-Doyle W Leow JJ Orsola A Chang SL Bellmunt J. Improving selection criteria for early cystectomy in high-gradeT1 bladder cancer: a meta-analysis of 15,215 patients. J Clin Oncol. (2015) 33:643–50. 10.1200/JCO.2014.57.6967 25559810

[73] Lawless M Gulati R Tretiakova M. Stalk versus base invasion in pT1 papillary cancers of the bladder: improved substaging system predicting the risk of progression. Histopathology. (2017) 71:406–14. 10.1111/his.13247 28470753 PMC5552491

[74] Leivo MZ Sahoo D Hamilton Z Mirsadraei L Shabaik A Parsons JK. Analysis of T1 bladder cancer on biopsy and transurethral resection specimens: comparison and ranking of T1 quantification approaches to predict progression to muscularis propria invasion. Am J Surg Pathol. (2018) 42:e1–10. 10.1097/PAS.0000000000000964 29076872

[75] Patriarca C Hurle R Moschini M Freschi M Colombo P Colecchia M. Usefulness of pT1 substaging in papillary urothelial bladder carcinoma. Diagn Pathol. (2016) 20:6. 10.1186/s13000-016-0466-6 26791567 PMC4721190

[76] Cheng L Weaver AL Neumann RM Scherer BG Bostwick DG. Substaging of T1 bladder carcinoma based on the depth of invasion as measured by micrometer: a new proposal. Cancer. (1999) 86:1035–43. 10.1002/(SICI)1097-0142(19990915)86:6:<1035::AID-CNCR20>3.0.CO 10491531

[77] Brimo F Wu C Zeizafoun N Tanguay S Aprikian A Mansure JJ. Prognostic factors in T1 bladder urothelial carcinoma: the value of recording millimetric depth of invasion, diameter of invasive carcinoma, and muscularis mucosa invasion. Hum Pathol. (2013) 44:95–102. 10.1016/j.humpath.2012.04.020 22939956

[78] Hu Z Mudaliar K Quek ML Gladell P Paner GP Barkan GA. Measuring the dimension of invasive component in pT1 urothelial carcinoma in transurethral resection specimens can predict time to recurrence. Ann Diagn Pathol. (2014) 18:49–52. 10.1016/j.anndiagpath.2013.11.002 24370460

[79] Kassouf W Spiess PE Brown GA Munsell MF Grossman HB Siefker-Radtke A. pT0 stage at radical cystectomy for bladder cancer is associated with improved outcome independent of traditional clinical risk factors. Eur Urol. (2007) 52:769–74. 10.1016/j.eururo.2007.03.086 17434254 PMC2691552

[80] Tilki D Svatek RS Novara G Seitz M Godoy G Karakiewicz PI. Stage pT0 at radical cystectomy confers improved survival: an international study of 4,430 patients. J Urol. (2010) 184:888–94. 10.1016/j.juro.2010.04.081 20643448

[81] Pignot G Houédé N Roumiguié M Audenet F Brunelle S Colin P. ypT0N0 after neoadjuvant chemotherapy and cystectomy for muscle-invasive bladder cancer: incidence and prognosis. A review from the bladder group of the French committee of oncology. Prog Urol. (2018) 28:567–74. 10.1016/j.purol.2018.07.003 30205925

[82] Boudreaux KJ Jr Clark PE Lowrance WT Rumohr JA Barocas DA Cookson MS. Comparison of american joint committee on cancer pathological stage T2a versus T2b urothelial carcinoma: analysis of patient outcomes in organ confined bladder cancer. J Urol. (2009) 181:540–6. 10.1016/j.juro.2008.10.038 19084855

[83] Tilki D Reich O Karakiewicz PI Novara G Kassouf W Ergün S. Validation of the AJCC TNM substaging of pT2 bladder cancer: deep muscle invasion is associated with significantly worse outcome. Eur Urol. (2010) 58:112–7. 10.1016/j.eururo.2010.01.015 20097469

[84] Yu RJ Stein JP Cai J Miranda G Groshen S Donald G. Superficial (pT2a) and deep (pT2b) muscle invasion in pathological staging of bladder cancer following radical cystectomy. J Urol. (2006) 176:493–9. 10.1016/j.juro.2006.03.065 16813876

[85] Ananthanarayanan V Pan Y Tretiakova M Amin MB Cheng L Epstein JI. Influence of histologic criteria and confounding factors in staging equivocal cases for microscopic perivesical tissue invasion (pT3a): an interobserver study among genitourinary pathologists. Am J Surg Pathol. (2014) 38:167–75. 10.1097/PAS.0000000000000096 24145655

[86] Mazzucchelli R Cheng L Lopez-Beltran A Scarpelli M Montironi R. Clinicopathological significance of lymphovascular invasion in urothelial carcinoma. Anal Quant Cytopathol Histpathol. (2012) 34:173–9. 23016463

[87] Tilki D Shariat SF Lotan Y Rink M Karakiewicz PI Schoenberg MP. Lymphovascular invasion is independently associated with bladder cancer recurrence and survival in patients with final stage T1 disease and negative lymph nodes after radical cystectomy. BJU Int. (2013) 111:1215–21. 10.1111/j.1464-410X.2012.11455.x 23181623

